# A N, S-Containing Graphene Oxide Composite for the Adsorptive Removal of p-Nitrophenol from Aqueous Solutions

**DOI:** 10.3390/molecules30092046

**Published:** 2025-05-04

**Authors:** Bi Yang, Tao-Tao Shi, Wei-Guo Hu, Guan-Jin Gao, Yi-Ping Liu, Jin-Gang Yu

**Affiliations:** 1 College of Chemistry and Chemical Engineering, Central South University, Changsha 410083, China; 222311019@csu.edu.cn (B.Y.); willhu1@126.com (W.-G.H.); 232302049@csu.edu.cn (G.-J.G.); 2 Scientific Research Academy of Guangxi Environmental Protection, Nanning 530022, China; t9121031@163.com; 3 Hunan Provincial Institute of Cotton and Sericultural Research, Hunan Academy of Agricultural Sciences, Changsha 410127, China

**Keywords:** graphene oxide, 3-amino-5-mercapto-1,2,4-triazole, adsorption, p-nitrophenol

## Abstract

A novel 3-amino-5-mercapto-1,2,4-triazole functionalized graphene oxide composite (GO-ATT) was successfully prepared via a covalent coupling method, then employed for the removal of p-nitrophenol (PNP) from wastewater. The morphology as well as the composition of GO-ATT composite were investigated using Fourier transform infrared spectroscopy (FT-IR), scanning electron microscopy (SEM), thermogravimetric analysis (TGA), X-ray diffraction spectroscopy (XRD), and X-ray photoelectron spectroscopy (XPS). The surface charge of GO-ATT composite was evaluated by Zeta potential analyses. The surface area and pore size distribution of GO-ATT composite were analyzed using specific surface analyses using the Brunauer–Emmett–Teller (BET) method. Batch adsorption experiments were performed to investigate the effects of conditional factors, including contact time, solution pH, initial PNP concentration, and contact temperature, on the adsorption process. A maximum adsorption capacity of PNP by GO-ATT composite (0.287 mmol g^−1^) could be obtained at 25 °C. Freundlich isotherm (*R*^2^ > 0.92505) can better describe the adsorption behavior of PNP on GO-ATT composite. The thermodynamic functions (Δ*G*°, Δ*H*°, Δ*S*°) indicate that adsorption is a spontaneous, endothermic, entropy-increasing process and features physisorption. The adsorption behavior of PNP on GO-ATT composite conformed to the nonlinear pseudo-second-order kinetic model. Adsorption mechanism investigation indicated that the electrostatic, π-π stacking, and hydrogen bonding interactions were involved in the adsorption process. After 10 adsorption–desorption cycles, the adsorbent exhibited a stable and efficient removal rate (94%) for PNP. Due to its advantages of a high efficiency, excellent reusability, and high stability, the covalently coupled GO-ATT composite might be used as an effective adsorbent for the removal of phenolic contaminants from wastewater.

## 1. Introduction

In recent years, a diverse range of organic pollutants have found their way into the aquatic environment as a result of swift global industrialization [1,2]. One of the main pollutants consists of phenolic compounds that are discharged from various industries including the petrochemical industry, petroleum refineries, coke ovens, and herbicides. Phenolic contaminants are chemically and microbially stable, causing them to persist and be widely dispersed in the environment. In addition, phenolic contaminants have negative effects on human beings and aquatic organisms even at very low concentrations [3]. For example, bisphenol A (BPA) is a carcinogen. It can also cause endocrine disruption [4]. The p-nitrophenol (PNP) in wastewater is also difficult to degrade naturally, leading to unusual accumulation in biological systems and non-negligible toxic and mutagenic hazards [5]. Developing effective, cost-saving, and simple methods to remove phenolic contaminants is therefore essential [6].

Various conventional techniques are available for removing phenolic contaminants from wastewater, including extraction [7], membrane filtration [8], photocatalysis [9], and adsorption [10]. Among them, due to its advantages such as its easy operation, easy regulation, high efficiency, low cost, and its time saving, the adsorption method has been extensively studied and widely used in practice [11,12,13]. Various adsorbents such as Fe_3_O_4_/ZIF-8 [14], steel slag [15], N-doped highly microporous carbon [16], granulated activated carbon (GAC) [17], mixed bio-waste of shrimp shell, and rice husk [18], among others, have been successfully implemented to remove phenol and p-nitrophenol (PNP) from contaminated water. While the previously reported adsorbents had relatively high adsorption capacity, they had a number of drawbacks, including poor selectivity, complex preparation steps, and high prices.

Graphene oxide (GO) is a product of chemical oxidation and exfoliation of graphite powder. It is rich in oxygen-containing functional groups (hydroxyl, carboxyl, and epoxy groups) on its surface and edges. [19]. The formation of hydrogen bonding, π-π stacking, and electron donor–acceptor (EDA) interactions with phenols can benefit their possible adsorption. However, the well-dispersed nature of GO makes its separation from aqueous solutions after adsorption difficult and tedious [20]. Therefore, the reservation of GO may cause extra environmental pollution [21,22]. Fortunately, the construction of GO-based composites has been proved to be a feasible methodology. In recent years, various GO-based composites have been developed as promising adsorbents for wastewater treatment [22,23,24,25]. Polyethylene glycol-functionalized reduced GO coupled with zinc oxide (rGO-PEG-ZnO) composite could be used for the efficient removal of various phenolic pollutants such as 2,4–dichlorophenol (2,4–DCP), phenol, 2–chlorophenol (2–CP), and bisphenol–A (BPA) [26]; the modification by polyacrylic acid of GO could introduce (C=O) groups on its surface, which would enhance the adsorption performances of the GO (GO-PAA) composite for phenol [27]. Chitosan-crosslinked GO (GO-CS) composite also exhibited enhanced adsorption performances toward cationic dyes in aqueous solutions; the pore occupation and electrostatic interaction contributed to the efficient adsorption [28]. Obviously, the heteroatomic groups, including nitrogen (N)- and oxygen (O)-containing groups, are beneficial to the removal of phenols.

Due to its sulfur (S)-containing functional group and triazole ring, 3-Amino-5-mercapto-1,2,4-triazole (ATT) was used to modify silica nanoparticles for the removal of Ag^+^ from an aqueous solution [29]. Although a high maximum adsorption capacity of 124.52 mg g^−1^ could be obtained due to the amino groups and heterocyclic π-electrons—which benefited the EDA and electrostatic interactions—the preparation procedures seemed to be tedious or complex. However, the covalent coupling of the abundant carboxyl groups (-COOH) of GO with this excellent modifier, ATT, might produce a versatile adsorbent for the removal of organic contaminants, and the fabrication process could be greatly simplified. In addition, the introduction of S-containing groups onto GO would improve its hydrophobic properties [30], which undoubtedly might facilitate its recycling after adsorption.

In this study, a novel GO-ATT composite was developed, in which ATT was covalently grafted onto the surface of GO via an amidation reaction to introduce more active adsorption sites. The composite was successfully applied to the removal of p-nitrophenol (PNP), and the effects of key parameters, such as the initial concentration of the solution, adsorption time, temperature, and pH, on the adsorption performance were systematically investigated. The adsorption mechanism of GO-ATT composites on PNP was deeply revealed by characterization means such as Fourier transform infrared spectroscopy (FT-IR) and zeta potential analysis.

## 2. Results and Discussions

### 2.1. Characterization of the Samples

#### 2.1.1. Scanning Electron Microscopy (SEM)

**Figure 1:**
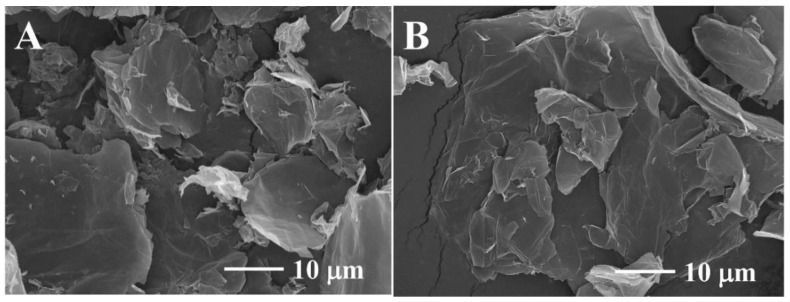
Scanning electron microscope (SEM) images of GO-ATT composites before (**A**) and after (**B**) adsorption of PNP.

The surface morphology of the GO-ATT composite pre- and post-adsorption of the PNP was analyzed by SEM (Figure 1), and the GO-ATT composite clearly retains the lamellar structure of GO (Figure 1A); the rougher surface due to the amidation reaction of ATT with the carboxyl groups on the surface of GO can be observed, indicating that a peculiar accumulation in the lamella occurs [31]. After adsorption of PNP, the GO-ATT composite (GO-ATT-PNP) was not significantly different from the original composite (Figure 1B).

**Figure 2:**
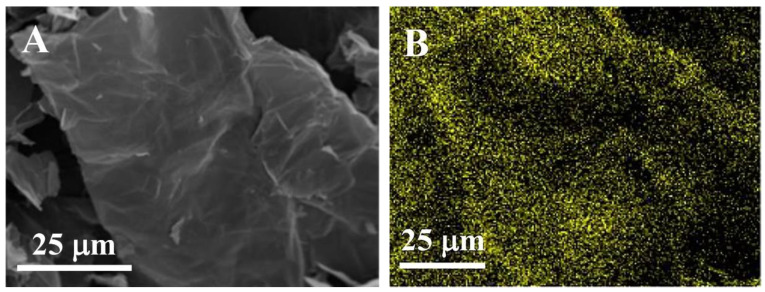

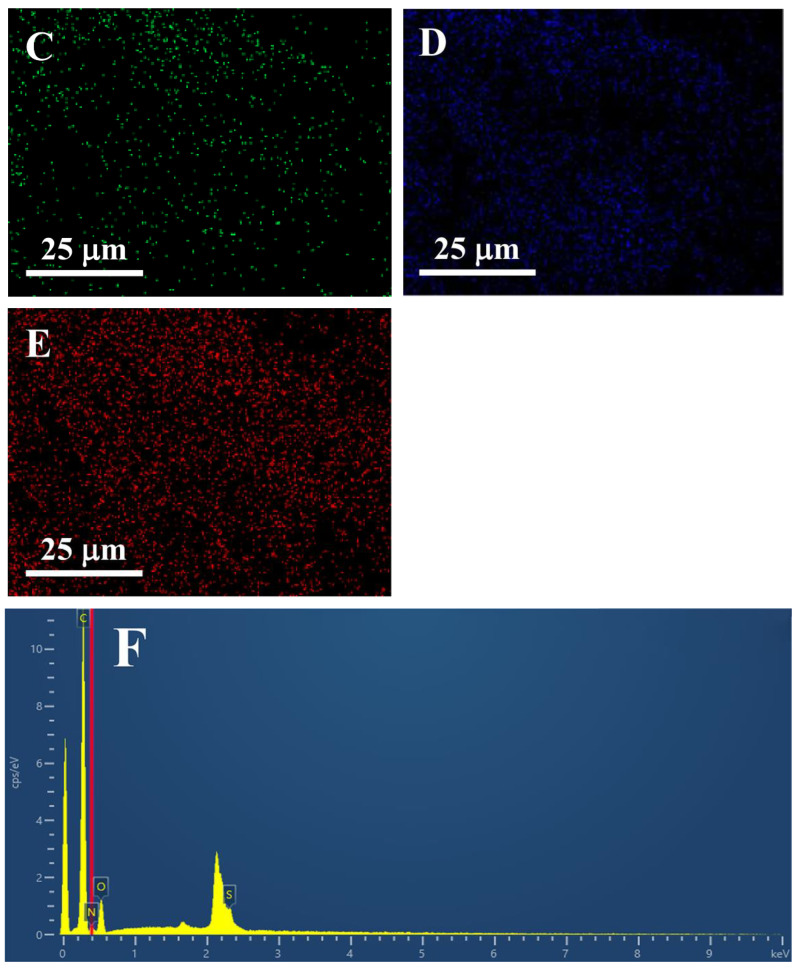
Elemental mapping images of GO-ATT: (**A**) the scanning field of view; (**B**) C; (**C**) N; (**D**) O; (**E**) S; (**F**) EDS of GO-ATT.

The elemental distribution and composition of GO-ATT composite were resolved by elemental mapping analysis and EDS (Figure 2). The uniform distribution of N and O elements on the composite surface might be beneficial to the adsorption of PNP (Figure 2A–E). C (18.1 wt.%), N (12.9 wt.%), O (48.3 wt.%), and S (20.7 wt.%) could clearly be detected, and the presence of N and S elements in the sample also confirms that ATT is successfully attached onto GO (Figure 2F).

#### 2.1.2. FT-IR Spectroscopy

**Figure 3:**
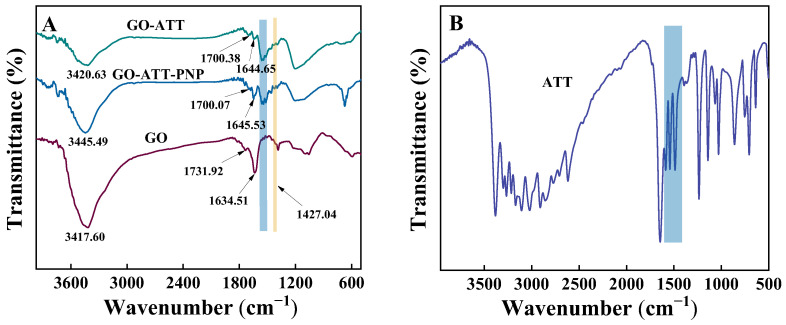
FTIR spectra of (**A**) GO and GO-ATT before and after PNP adsorption; (**B**) ATT.

**Figure 4:**
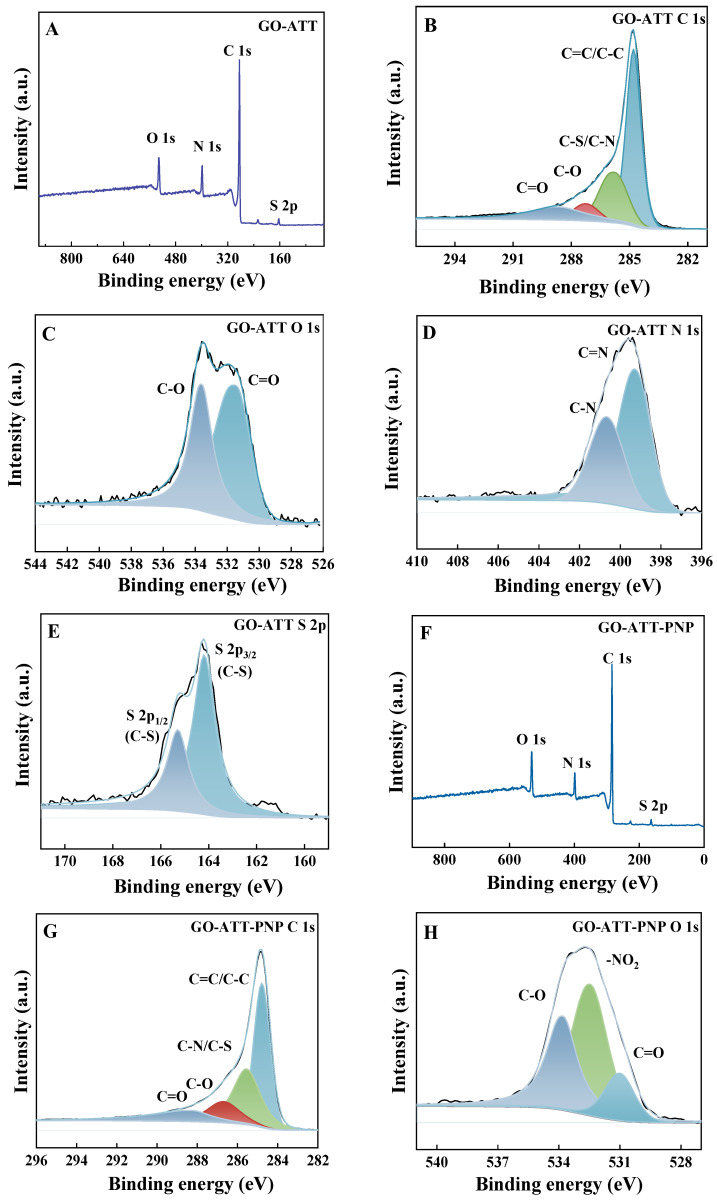

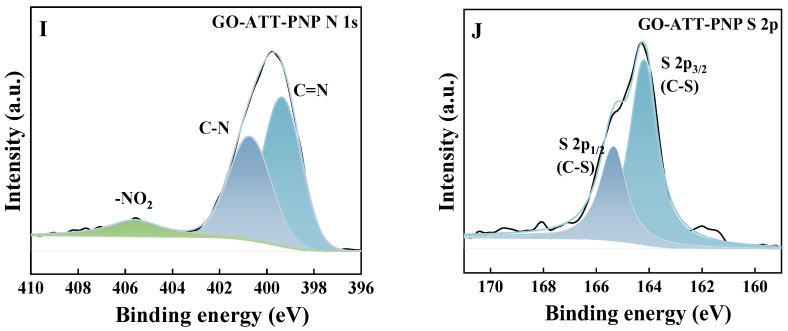
XPS spectra of GO-ATT composite: (**A**) survey spectra and split-peak fitting analysis of C 1s (**B**); O 1s (**C**); N 1s (**D**); and S 2p (**E**). XPS spectra of GO-ATT-PNP: (**F**) survey spectra; split-peak fitting analysis of C 1s (**G**); O 1s (**H**); N 1s (**I**); and S 2p (**J**).

The appearance of new absorption peaks and the shift of absorption peaks can be utilized to evaluate the chemistry of the surface functional groups. Figure 3 shows the FT-IR spectra of GO, ATT, GO-ATT, and GO-ATT-PNP. The absorption peaks at 3417.60 cm^−1^ and 1731.92 cm^−1^ are attributed to the O-H and C=O (carboxyl group) stretching vibrations of GO [32,33], respectively. For the GO-ATT composite, the absorption peaks at 3420.63 cm^−1^ and 1700.07 cm^−1^ correspond to the O-H/N-H and C=O stretching vibrations, respectively [34,35,36]. Compared with the FT-IR spectra of ATT (Figure 4B) and GO (Figure 4A), the GO-ATT composite possesses not only the characteristic peaks of ATT, but also a new absorption peak at 1427.04 cm^−1^. The newly appeared absorption peak at 1427.04 cm^−1^ can be attributed to the C-N stretching vibration in the amide bond. The successful preparation of GO-ATT composite is also confirmed by the stretching vibration peaks of C=O, which shifted to a lower wavenumber of 1700.38 cm^−1^. After the adsorption of PNP, the absorption peaks of O-H/N-H and the aromatic skeletal stretching vibrations shift from 3420.63 cm^−1^ and 1644.65 cm^−1^ to 3445.49 cm^−1^ and 1645.53 cm^−1^, respectively, suggesting that the hydrogen bonding and π-π stacking interactions between PNP and GO-ATT composite occur [37,38].

#### 2.1.3. XPS

In order to analyze the surface properties of the materials before and after adsorption, GO-ATT and GO-ATT-PNP were characterized by using XPS (Figure 4, Table 1). The presence of -NO_2_ in GO-ATT-PNP confirmed the successful adsorption of PNP by the GO-ATT composite, and the existence of hydrogen bonding and π-π stacking interactions between the GO-ATT composite and PNP is further confirmed by the changes in the binding energies of the C-O/C-N and C=O bonds before and after adsorption [39].

#### 2.1.4. Nitrogen Adsorption–Desorption Isotherms

**Figure 5:**
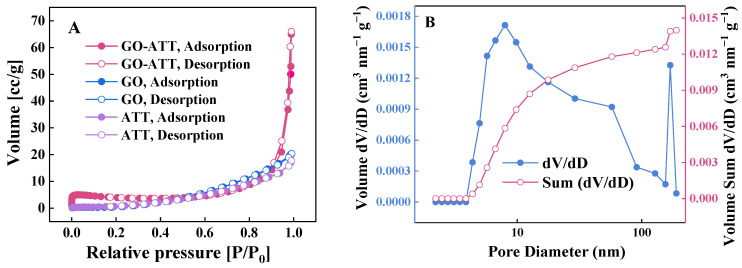
GO-ATT composite: (**A**) N_2_ adsorption/desorption isotherms; (**B**) pore volume distribution.

The specific surface area of GO, ATT, and GO-ATT composite as well as the pore size distribution of GO-ATT composite were determined using N_2_ adsorption–desorption isotherms (Figure 5). The results showed that the specific surface area, cumulative pore volume and average pore diameter of GO-ATT composite were 20.61 m^2^ g^−1^, 0.103 cm^3^ g^−1^, and 9.91 nm, respectively. The specific surface areas of GO and ATT were 1.15 m^2^ g^−1^ and 1.72 m^2^ g^−1^, respectively. Previous studies have shown that the specific surface area of GO ranges from 2 to 1000 m^2^ g^−1^, but the specific surface area of pure GO is slightly lower in practical tests, which is mainly caused by the severe agglomeration between the dried GO lamellae [40,41]. The graft of ATT onto the GO surface by the covalent-coupling strategy not only effectively suppressed the agglomeration behavior of GO, but also introduced abundant functional groups onto the surface, which further improved the adsorption selectivity of the composite material.

#### 2.1.5. TGA

**Figure 6:**
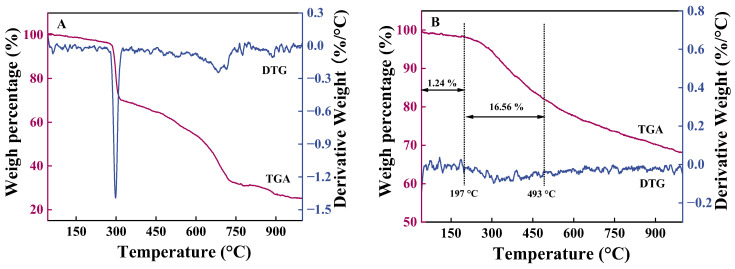
TGA curves of the samples: (**A**) ATT; (**B**) GO-ATT composite.

The thermal stability of the ATT molecule and GO-ATT composite was investigated by TGA under an air atmosphere, from room temperature to 1000 at a ramp rate of 10 °C min^−1^. Figure 6 illustrates the TGA and DTG curves of the ATT molecule and GO-ATT composite. ATT and GO-ATT show a small weight loss at temperatures below 197 °C. This is due to the evaporation of water [42]. The maximum weight loss of the GO-ATT composite occurs in the temperature range of 197–493 °C, which is attributed to the decomposition of specific functional groups in the composite, and the subsequent weight loss can be attributed to the cleavage of the carbon skeleton. In addition, the DTG curves indicate that the maximum weight loss of the composite occurs around 300 °C, which exhibits excellent thermal stability.

#### 2.1.6. XRD

**Figure 7:**
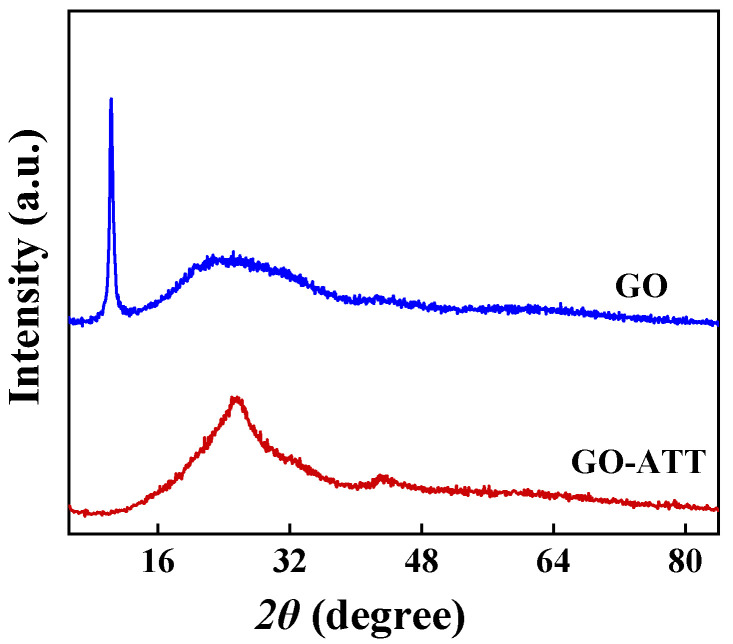
XRD patterns of GO, GO-ATT.

The XRD patterns of GO and GO-ATT are shown in Figure 7. GO exhibits two major characteristic peaks at 2θ of 10.30° and 23.81°, and the interlayer spacing of GO is calculated to be 0.866 nm according to the Bragg’s equation, which is larger than that of the graphite (2θ = 26.5°, 0.34 nm) [43]. Compared with the XRD pattern of GO, the characteristic peaks of GO-ATT reveal significant shifts, indicating that ATT was successfully grafted onto GO to form new composite crystals [44].

### 2.2. Process Optimization and Adsorption Modeling

#### 2.2.1. Adsorption Performances

**Figure 8:**
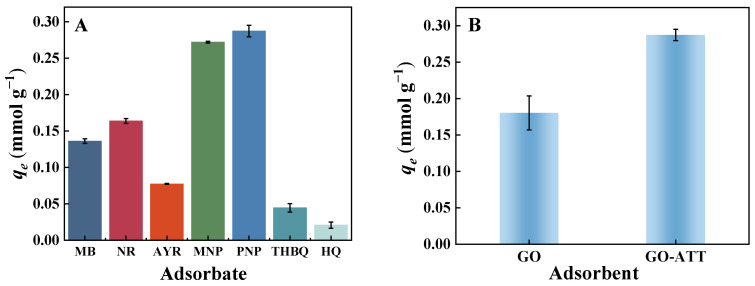
(**A**) Adsorption performance of GO-ATT composite toward dyes and phenols (m_adsorbent_ = 5.0 mg, *C*_0_ = 50 mg L^−1^, t = 120 min, *T* = 25 °C). (**B**) Adsorption performance comparison between GO and GO-ATT for PNP removal.

The adsorption amounts of GO-ATT composites for MB, NR, AYR, MNP, PNP, THBQ, and HQ were 0.136, 0.164, 0.077, 0.272, 0.287, 0.044, and 0.021 mmol g^−1^, respectively (Figure 8A). Among them, the adsorption of PNP by the GO-ATT composite was relatively higher, which was 1.59 times higher than that of unmodified GO (Figure 8B). The modification of ATT with GO not only improved the hydrophobicity of the composites, but also enhanced its adsorption performance for PNP. Remarkably, the adsorption capacity of the GO-ATT composite for PNP was 40 mg g^−1^ (0.287 mmol g^−1^), which was lower than some previously reported adsorbents (Table 2). However, a significantly lower initial concentration (50 mg L^−1^) and smaller dosage (0. 25 g L^−1^) of adsorbent were employed. In the subsequent experiments, PNP would be utilized as a model pollutant to investigate the possible interaction mechanisms between the adsorbent and adsorbate.

#### 2.2.2. Effect of Adsorption Conditions

##### Effect of Contact Time

**Figure 9:**
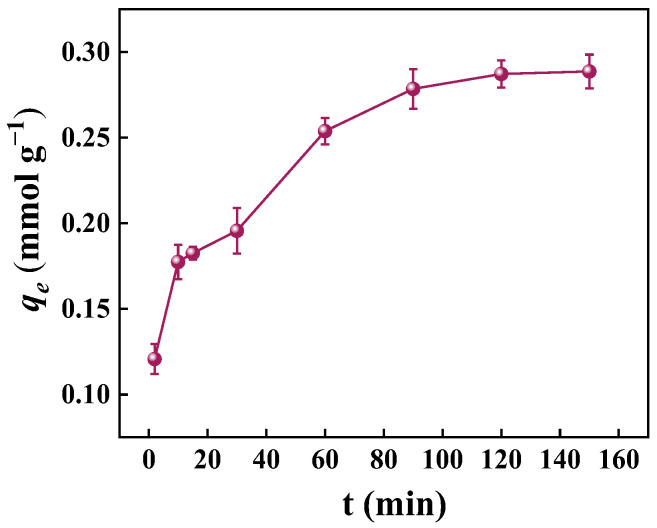
Effect of contact time on removal of PNP by GO-ATT composite.

Figure 9 shows the effect of contact time on the adsorption of PNP by GO-ATT composite. The adsorption experiments were carried out at 25 °C with an initial PNP concentration of 50 mg L^−1^. The results show that the adsorption of PNP by GO-ATT reached the adsorption equilibrium at 120 min. PNP adsorbed on GO-ATT can be divided into two phases: In the initial stage (t = 0–60 min), the concentration difference between the native solution and the surface of the adsorbent was large, which provided the driving force for the diffusion of PNP, and a large amount of PNP diffused onto the surface of the adsorbent and interacted with the active sites to produce rapid adsorption [50]. In the second stage (60–120 min), the active sites exposed by the adsorbent were gradually occupied [51], the concentration of PNP decreased, the diffusion rate decreased, and the adsorption process gradually slowed down, reaching equilibrium at about 120 min [52], when the adsorption of PNP by GO-ATT reached a maximum value of 0.287 mmol g^−1^.

##### Effect of pH

**Figure 10:**
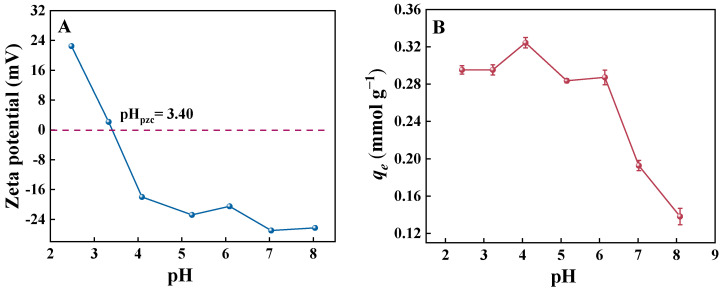
(**A**) Zeta potential of GO-ATT composite; (**B**) effect of initial solution pH (*C*_0_ = 50.0 mg L^−1^, t = 120 min, *T* = 25 °C).

The pH of the solution has a significant effect on the adsorption process; in particular, under different pH conditions, protonation and deprotonation occur on the adsorbent surface, resulting in a change in the surface charge distribution; in addition, the adsorbate will have different chemical forms according to the acidity and alkalinity of the solution, which will affect the adsorbate–adsorbent interaction [12]. Therefore, in this study, the PNP solution was adjusted to different pH values, including 2.43, 3.23, 4.08, 5.15, 6.14, 7.02, and 8.09, to investigate the effect of pH on the adsorption of p-nitrophenol by the GO-ATT composite, and the results are shown in Figure 10B, which shows that the adsorption of PNP by GO-ATT composite showed an initial increase and then a decreasing trend. Given PNP’s pKa (=7.15), it is predominantly molecular at pH < 7.15 and may be positively charged at a low pH, whereas it is predominantly anionic at pH > 7.15 [53]. Obviously, the GO-ATT composite would be positively charged when the pH of the solution is below pH_pzc_ (Figure 10A), which creates the possibility of electrostatic repulsion toward the protonated PNP, causing low-level adsorption. When the solution pH > pH_pzc_, the deprotonation of the GO-ATT composite occurs. That is, the GO-ATT composite is negatively charged, which favors the adsorption of PNP; thus, the amount of adsorption increases in the pH range 3.0 to 4.0 (Figure 10B). As the solution pH continues to increase, PNP is gradually deprotonated and the adsorption capacity of the GO-ATT composite decreases due to the emerging electrostatic repulsions. Undoubtedly, the electrostatic forces play an important role in the adsorption process.

##### Adsorption Kinetics

Nonlinear pseudo-first-order and nonlinear pseudo-second-order kinetic models were used to fit the experimental data to determine the mechanism of the GO-ATT composite for PNP. The results are presented in Figure S1 and Table S1. The coefficient of determination (*R*^2^) obtained from the nonlinear pseudo-second-order model is higher than that of another model, and the adsorption capacity calculated by this model is closer to the experimental data. Therefore, the nonlinear pseudo-second-order kinetic model can better describe the adsorption behavior of PNP on the GO-ATT composite.

##### Adsorption Isotherms

**Figure 11:**
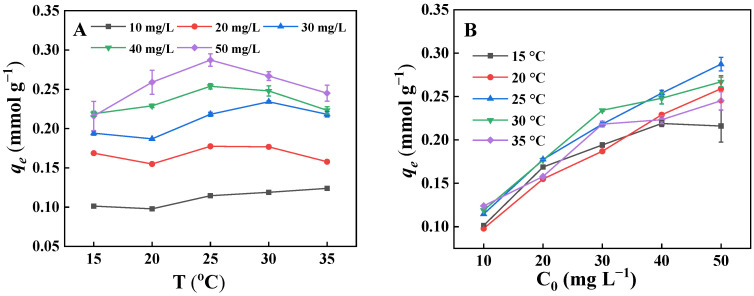
Adsorption properties of GO-ATT composite for PPNP: effect of temperature (**A**) and initial PNP concentration (**B**).

To uncover the adsorption mechanism, the impacts of initial PNP concentration and temperature on the adsorption of PNP by the GO-ATT composite were examined (Figure 11). As the PNP concentration increases at a given temperature, the adsorption capacity of the GO-ATT composite for PNP increases. The increase in adsorption capacity is closely related to the larger mass transfer effect; that is, the increase in initial concentration might cause more PNP to diffuse from the solution to the surface of an adsorbent, which undoubtedly accelerates the adsorption process.

Adsorption isotherms are used to study the adsorption of adsorbate molecules onto the adsorbent [54]. In this study, the experimental data of the GO-ATT composite for the adsorption of PNP were fitted using the Langmuir and Freundlich models.

The results are shown in Figure S2 and Table S2. The higher *R^2^* of the Freundlich model indicates that the adsorption of PNP by the GO-ATT composite is heterogeneous [55,56]. From the data presented in Table S2, it can be seen that the value of n is greater than 1 and the value of 1/n is between 0 and 1, indicating that the adsorption of PNP by the GO-ATT composite is a physical process, which is consistent with the results discussed earlier [57].

##### Adsorption Thermodynamics

The influence of temperature on the adsorption of PNP on the GO-ATT composite has been investigated by the calculation of thermodynamic parameters. The results are shown in Figure S3 and Table S3. The positive Δ*H*° (14.492 kJ mol^−1^) falls within the range of 2.1 to 20.9 kJ mol^−1^, indicating that the adsorption of PNP onto the GO-ATT composite is an endothermic and physisorption process [58,59,60]. The entropy becomes positive (Δ*S*° = 56.5754 J K^−1^ mol^−1^), implying that adsorption is entropy-increasing [61]. The negative values of Δ*G*° within the range of −20 to 0 kJ mol^−1^ at all tested temperatures suggest that the adsorption of PNP by GO-ATT composites is a spontaneous physisorption process [62,63].

### 2.3. Reusability of GO-ATT

To assess the reusability of the GO-ATT composite, a series of 10 consecutive adsorption–desorption experiments were carried out. Specifically, 20.0 mL of PNP solution (10 mg L^−1^) was mixed with 50.0 mg of GO-ATT composite. The mixture was shaken at 25 °C for 120 min. Subsequently, 40.0 mL of ethanol was used to desorb PNP from the GO-ATT composite, which was then directly used in the next adsorption–desorption cycle. The adsorption–desorption processes were repeated 10 times.

**Figure 12:**
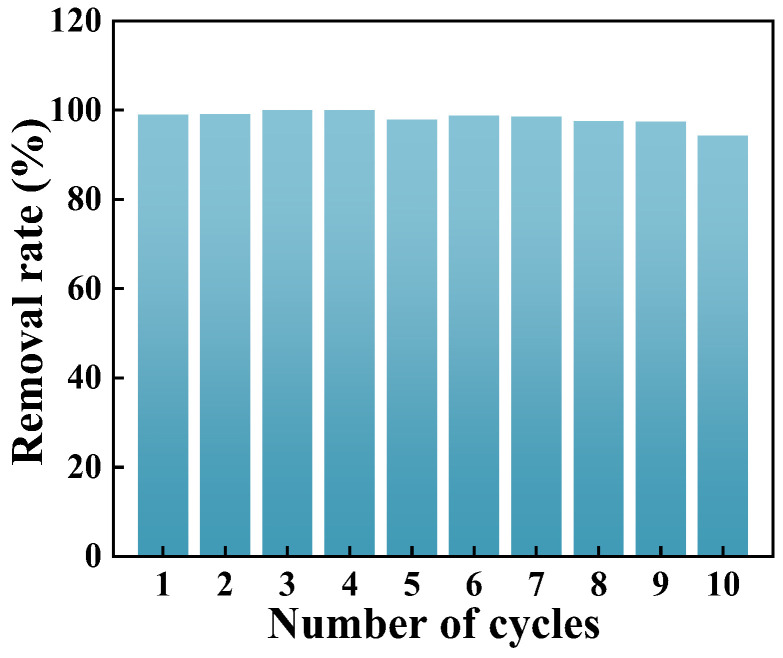
Reuse performance of GO-ATT.

As depicted in Figure 12, following 10 adsorption–desorption cycles, the removal rate of PNP could still attain 94%, signifying that the GO-ATT composite exhibits excellent reusability. Consequently, GO-ATT composite can be employed as a promising and sustainable adsorbent.

### 2.4. Adsorption Mechanism

**Figure 13:**
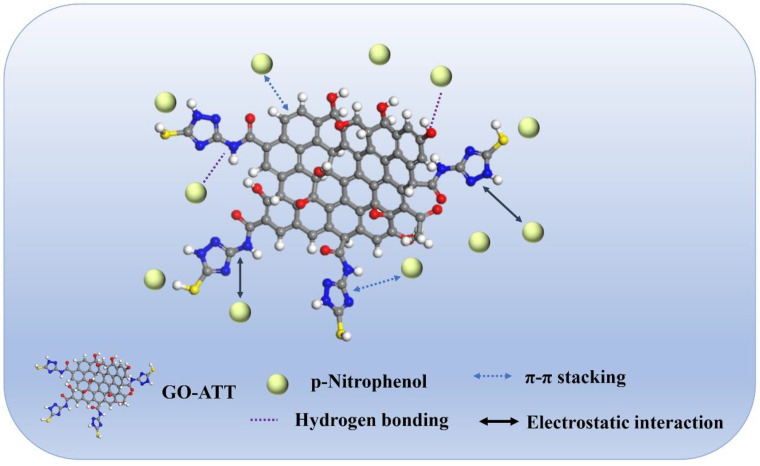
Mechanism of adsorption.

The GO-ATT composite is rich in N/O functional groups, which can form hydrogen bonds with PNP. Additionally, the -NH- and -OH groups on the surface of the GO-ATT composite would be protonated or deprotonated by varying the initial solution pH, thus altering the charge distributions on the surface of the adsorbent and creating electrostatic interactions with the adsorbate. The aromatic π-electrons on the GO-ATT composite can form a π-π stacking effect with the aromatic skeleton of phenols to enhance the adsorption process [64].These intermolecular interactions could be confirmed by FT-IR spectra, XPS, and elemental mapping analyses, as previously discussed. Based on these analyses, a schematic diagram of the proposed adsorption mechanism is presented (Figure 13).

## 3. Methodology

### 3.1. Preparation of GO-ATT Composite

**Figure 14:**
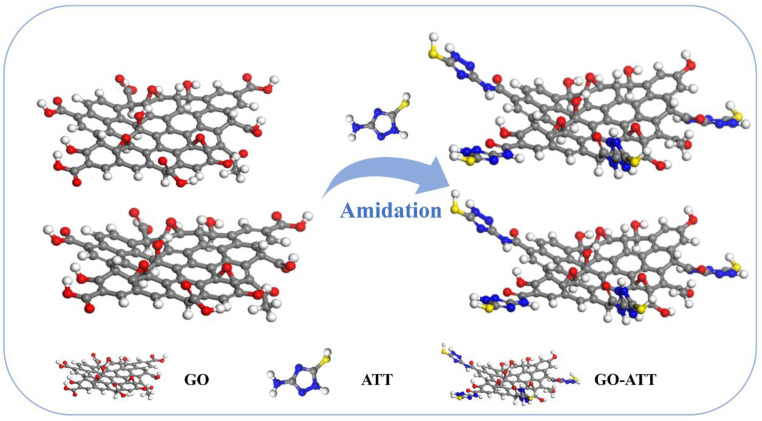
Synthesis of GO-ATT composite schematic.

GO-ATT composite was prepared by the amidation reaction between the carboxyl groups of GO and the amino group on ATT (Figure 14), and 1.0 g of GO was added in a 250 mL round-bottom flask, to which 90 mL of DMF solution was added and sonicated for 30 min to ensure that GO was uniformly dispersed in the DMF solution. Then, 2.0 g ATT, 0.15 g DCC, and 0.15 g DMAP were added, stirred for 2 min, and sonicated for a further 30 min. The mixture was heated to 160 °C. It was allowed to react for 26 h under constant magnetic stirring. After the reaction, the mixture was allowed to cool naturally to room temperature. It was then filtered and washed repeatedly with ethanol and ultrapure water. Finally, the residual solid was dispersed in ultrapure water, snap-frozen at −50 °C, and lyophilized to obtain the GO-ATT composite.

### 3.2. Characterization of Materials

GO-ATT composite was systematically characterized using multiple analytical techniques. Scanning electron microscopy (SEM) was employed to observe the changes in surface morphology of the composite before and after the adsorption of phenols. Fourier transform infrared spectroscopy (FT-IR) was utilized to identify the functional groups in the composite and monitor their variations during the adsorption process. X-ray photoelectron spectroscopy (XPS) was applied to analyze the surface elemental composition and chemical states of the composite. The structure of the composite was examined by X-ray diffraction (XRD). The surface charge characteristics under different pH conditions were investigated through Zeta potential analysis. The specific surface area and pore size distribution were determined by N_2_ adsorption–desorption using the Brunauer–Emmett–Teller (BET) method. Additionally, the thermal stability and decomposition behavior of the composite were studied by thermogravimetric analysis (TGA).

### 3.3. Adsorption Experiments

A series of adsorption experiments were carried out to evaluate the adsorption performance of GO-ATT composite. The experiments were all carried out in 100 mL conical flasks by adding 5.000 mg of the prepared GO-ATT adsorbent and 20.00 mL of p-nitrophenol (PNP) solution with a specific initial concentration, which were subsequently placed in a thermostatic oscillator for a certain period of time. The effects of several conditional parameters on the adsorption performance of PNP by the GO-ATT composite were systematically investigated. The equilibrium time was determined by extracting and analyzing the solution sample at different time intervals (2, 5, 10, 15, 30, 60, 90, 120, and 150 min) during the adsorption process at 25 °C. The effect of initial pH values (2–8) on the adsorption was investigated at 25 °C. The effect of initial PNP concentration (10, 20, 30, 40, and 50 mg L^−1^) and adsorption temperature (25, 30, 35, 40, and 45 °C) on the adsorption process was investigated at the natural pH of the PNP solution. All adsorption experiments were performed three times to show the range of errors of the experimental results.

After adsorption, the mixture was filtered using a 0.45 μm microporous filter membrane. The obtained filtrate was appropriately diluted and the absorbance value was determined by UV-Vis spectrophotometry, and the adsorbate concentration after adsorption could be determined according to the standard curve. All experimental data were presented in the form of the mean and standard deviations. The equilibrium adsorption capacity (*q_e_*, mmol g^−1^), adsorption capacity (*q_t_*, mmol g^−1^), and removal rate (R, %) of GO-ATT for different adsorbates could be calculated using the Equations (S1)–(S3).

Reusability experiments were carried out as follows: 50.00 mg of adsorbent and 20.00 mL of PNP solution at a concentration of 10.00 mg L^−1^ were added to a 100 mL conical flask, which was placed in a thermostatic oscillator and oscillated for 120 min at 25 °C to ensure that the adsorption process reached equilibrium. After PNP adsorption, the mixture was filtered, and the adsorbent on the filter paper was washed using ultrapure water. The collected adsorbent was used in the desorption process. Briefly, 40.00 mL of ethanol was used as the eluent, and the desorption was carried out for 120 min to ensure that PNP was fully desorbed from the adsorbent. After desorption, the mixture was filtered, then washed with ultrapure water. The adsorbent on the filter paper was collected and directly used in the next adsorption–desorption cycle. The adsorption–desorption process was repeated 10 times to evaluate the reusability of the adsorbent.

## 4. Conclusions

A novel GO-ATT composite was rationally developed by the covalent coupling method. Its physicochemical properties were analyzed by different characterization methods. The GO-ATT composite showed an adsorption capacity of 0.287 mmol g^−1^ for PNP. The electrostatic, π-π stacking, and hydrogen bonding interactions were involved in the adsorption process. The nonlinear pseudo-second-order kinetic model and Freundlich isothermal model could better describe the adsorption behaviors of PNP on the GO-ATT composite. The adsorption–desorption experiments showed that the prepared GO-ATT composite might possess good reusable properties. Results from this study suggest that the GO-ATT composite is a potential adsorbent for phenolic contaminant removal from wastewater.

## Figures and Tables

**Table 1:** Surface element composition of GO-ATT and GO-ATT-PNP.

Samples		GO-ATT	GO-ATT-PNP
		Binding Energy (eV)	
C	C=C/C-C	284.80	284.80
	C-S/C-N	285.78	285.55
	C-O	287.25	286.68
	C=O	288.73	288.27
O	C-O	533.59	533.79
	C=O	531.47	531.01
	-NO_2_	-	532.48
N	C=N	399.28	399.36
	C-N	400.67	400.72
	-NO_2_	-	405.59
S	C-S (S 2p_3/2_)	164.19	164.16
	C-S (S 2p_1/2_)	165.29	165.31

**Table 2:** Comparison of adsorption capacity of GO-ATT with other adsorbents for PNP adsorption.

Adsorbent	Adsorbent Dosage	*C*_0_ (mg L^−1^)	q_max_ (mg g^−1^)	Ref.
CGA	0.015 g/0.015 L	5000	46.8	[17]
MPC	0.75 g/L	26	31.15	[45]
FA@PNP-SMIP	0.01 g/0.05 L	160	102.5	[46]
Steel slag	0.02 g/0.025 L	100	109.66	[15]
10% Fe-MCA	0.002 g/0.02 L	250	141	[47]
DNNH	0.6 g/L	15	22.10	[48]
CD-MIP	0.01 g/0.025 L	50	26.87	[49]
GO-ATT	0.005 g/0.02 L	50	40	This work

Notes: CGA: Commercial granulated activated carbon DARCO; MPC: synthesized by direct pyrolysis of the metal–organic framework MIL-53 (Fe) at 600 °C; FA@PNP-SMIP: microwave-assisted fly ash@p-nitrophenol surface molecularly imprinted polymer; 10% Fe-MCA: mass ratio of Fe and resorcinol formaldehyde xerogel at 1:10 ratio; DNNH: double network nanocomposite hydrogel; CD-MIP: DMAEMA-grafted cellulose-based molecularly imprinted polymer.

## Data Availability

Data are contained within the article and Supplementary Materials.

## Supplementary Materials

The following supporting information can be downloaded at https://www.mdpi.com/article/10.3390/molecules30092046/s1, Figure S1: (A) Fitted adsorption kinetic curves by nonlinear pseudo-first-order models; (B) Fitted adsorption kinetic curves by nonlinear pseudo-second-order models. Figure S2: (A) The fitting curves of Langmuir isotherm model; (B) The fitting curves of Freundlich isotherm model. Figure S3: Experimental data and fitted curves of lnk_d_ versus 1/T calculated from the van der Waals diagram of GO-ATT composites adsorbed PNP. Table S1: Adsorption kinetic parameters for the adsorption of PNP onto GO-ATT composites. Table S2: Adsorption isothermal parameters for the adsorption of PNP onto GO-ATT composites. Table S3: Thermodynamic parameters of the adsorption of PNP onto GO-ATT composites.
