# Supplementary material for: A N, S-Containing Graphene Oxide Composite for the Adsorptive Removal of p-Nitrophenol from Aqueous Solutions
Source: Molecules. 2025 May 4;30(9):2046. doi: 10.3390/molecules30092046 (PMC12073352; doi:10.3390/molecules30092046)
Supplement: Supplementary file 1 [file molecules-30-02046-s001.zip › molecules-3564172-supplementary.pdf]

Supporting Information

1. Chemicals

Flake graphite (80-90.5 wt.%) was supplied by Qingdao Braide Graphite Co., Ltd. (Qingdao, China). Concentrated sulfuric acid (H_2SO_4, 98.0 wt.%) and hydrochloric acid (HCl, 37.0 wt.%) were provided by Chengdu Colon Chemicals Co., Ltd. (Chengdu, China). Potassium permanganate ($KMnO_4$), Hydrogen peroxide (H_2O_2, 30.0 wt.%), phosphoric acid (H_3PO_4, 85% wt.%), hydroquinone (HQ), 3-nitrophenol (MNP), N, N-dimethylformamide (DMF) and PNP were purchased from Sinopharm Group Chemical Reagent Co., Ltd. (Shanghai, China). Methylene blue (MB, 98.5% wt.%) was produced from Beijing Chemical Factory. Alizarin Yellow R (AYR, 90% wt.%) was purchased from Hunan Xiangzhong Chemical Co. Ltd. (Shaoyang, China). Neutral Red (NR, 98% wt.%) was purchased from Tianjin Fuchen Chemical Reagent Co. Ltd. (Tianjin, China). Tert-butylhydroquinone (TBHQ, 98.0 wt.%), N, N'-Dicyclohexylcarbodiimide (DCC, 98.0 wt.%), 4-Dimethylaminopyridine (DMAP, 99.0 wt.%) and 3-amino-5-mercapto-1,2,4-triazole (ATT, 98.0 wt.%) were acquired from Aladdin Chemistry Co., Ltd. (Shanghai, China). All chemicals used in this study were of analytical grade and could be used without further purification.

2. Fabrication of GO

Preparation of GO using previously reported method was implemented，Briefly, 0.6 g of flake graphite and 3.0 g of $KMnO_4$ were added to a mortar and ground until a uniformly-dispersed mixture was obtained, which was transferred to a 250 mL round bottom flask. A mixed strong acid (8 mL of H_3PO_4, 72 mL of concentrated H_2SO_4,

volume ratio= 1: 9) was slowly dropwise added to the mixture under vigorous magnetic stirring and ice-water cooling. After the mixture appeared dark green, then it was heated to 50 °C and stirred for 12 h until the color changed to purple. The mixture was naturally cooled to room temperature, then transferred to a beaker containing ice-water under stirring. H_2O_2 was dropwise added to the mixture until it changed from purple to light yellow and no bubbles would be generated. Finally, the resulting mixture was centrifuged (10,000 rpm, 20 min), then washed continuously with 1 mol L^{-1} HCl (10,000 rpm, 20 min) and ultrapure water (10,000 rpm, 40 min) repeatedly until the supernatant was neutralized. The residual solid was collected and transferred to a beaker, diluted using ultrapure water, and freeze-dried to obtain sponge-like GO powder.

3. Characterization of the samples

SEM (JEOL, JSM-6360LV; Tokyo, Japan) was used to observe the surface morphology of GO-ATT composite pre- and post-adsorption of PNP. The structure of the composite was examined by XRD (Bruker AXS GmbH; D8 ADVANCE; Karlsruhe, Germany). XPS (Thermo Scientific ESCALAB Xi+, USA) and FT-IR (Shimadzu IR Prestige 21; Japan) were used to analyze the functional groups, chemical composition, and chemical state of GO-ATT composite pre- and post-adsorption of PNP. Zeta potential of GO-ATT composite as a function of pH was measured by a Zetasizer Nano ZS (Malvern Instruments Ltd.; UK). BET method was used to analyze the specific surface area and pore size of GO-ATT composite on a Kubo X1000 surface area analyzer (Beijing Builder Electronic Technology Co., LTD.; Beijing, China). The thermal stability of ATT, GO-ATT composite and GO-ATT-PNP was investigated by

TGA on a PerkinElmer instrument (Pyris 1PT-1600; USA) in the temperature range room temperature-1000 °C under air atmosphere at a heating rate of 10 °C min^{-1}.

4. Adsorption experiments

The absorbance (abs.; a.u.) at wavelengths of 664 nm for MB, 373 nm for AYR, 542 nm for NR, 318 nm for PNP, 221 nm for HQ, 289 nm for THBQ, and 327 nm for MNP was measured, respectively.

Equilibrium adsorption capacity (q_e, mmol g^{-1}) and removal rate (R, %) of GO-ATT for different adsorbates can be calculated using the equations **S1**, **S2**, and **S3**.

$$q_e = \frac{(C_0 - C_e)\cdot V}{m} \quad \textbf{(S1)}$$

$$R = \frac{C_0 - C_e}{C_0} \times 100\% \quad \textbf{(S2)}$$

$$q_t = \frac{(C_0 - C_t)\cdot V}{m} \quad \textbf{(S3)}$$

where q_e (mmol g^{-1}), C_0 (mmol L^{-1}), C_e (mmol L^{-1}), V (L), m (g), q_t (mmol g^{-1}), C_t (mmol L^{-1}), and R (%) represent the equilibrium adsorption capacity of GO-ATT composite, the initial adsorbate concentration, and the adsorbate concentration at equilibrium, the volume of adsorbate solution, the mass of GO-ATT composite, the adsorption capacity of GO-ATT composite at time t, the adsorbate concentration at time t and the removal rate, respectively.

5. Adsorption kinetics

Two couples of kinetic models were used to fit the experimental data of contact time on PNP adsorbed onto GO-ATT. The nonlinear pseudo-first-order and nonlinear pseudo-second-order models are expressed as follows:

nonlinear pseudo-first-order models:

$$q_t = q_e(1-e^{-k_1 t}) \quad \textbf{(S4)}$$

nonlinear pseudo-second-order models:

$$q_t = \frac{k_2 q_e^2 t}{1 + k_2 q_e t} \quad \textbf{(S5)}$$

where q_e (mmol g^{-1}) and q_t (mmol g^{-1}) are the adsorption capacity of the adsorbent for the target molecule at equilibrium and at time *t*, respectively; *t* (min) is the adsorption time; and k_1 (min^{-1}) and k_2 (g $mmol^{-1}$ min^{-1}) are the adsorption rate constants for the nonlinear pseudo-first-order model and the nonlinear pseudo-second-order model, respectively.

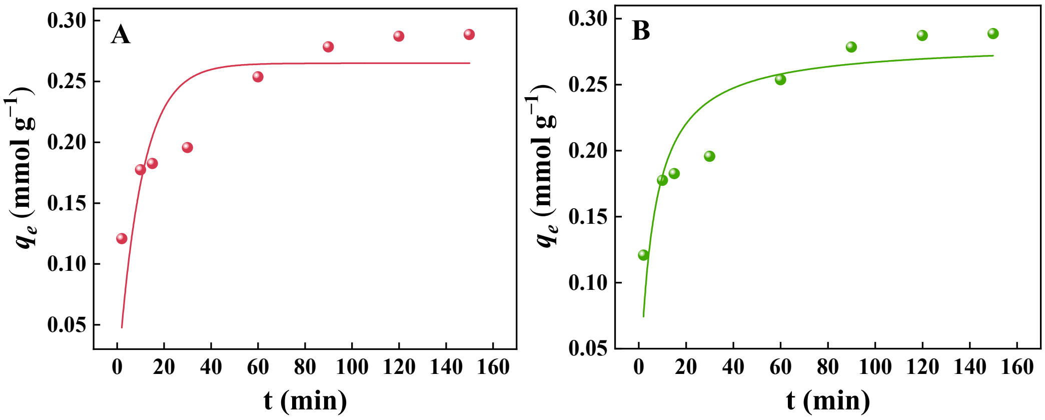

Fig. S1 (A) Fitted adsorption kinetic curves by nonlinear pseudo-first-order models; (B) Fitted adsorption kinetic curves by nonlinear pseudo-second-order models.

Table S1 Adsorption kinetic parameters for the adsorption of PNP onto GO-ATT composites.

Fitted parameter	Nonlinear pseudo-first-order models	Nonlinear pseudo-second-order models
R^2	0.61931	0.80845
k_1(min^{-1})	0.09842	--
k_2(min^{-1})	--	0.63048
q_e(mmol g^{-1})	0.26497	0.28211

6. Adsorption isotherms and thermodynamics

Two thermodynamic models were used to fit the experimental data of PNP adsorption on GO-ATT composites.

Langmuir model:

$$q_e = \frac{q_m K_L C_e}{1 + K_L C_e} \quad \textbf{(S6)}$$

Freundlich model：

$$q_e = K_F C_e^{\frac{1}{n}} \quad \textbf{(S7)}$$

where C_e (mmol L^{-1}) is the equilibrium concentration; q_e (mmol g^{-1}) is t is the amount of adsorption per unit mass of adsorbent at adsorption equilibrium; K_L (L $mmol^{-1}$) is the Langmuir equilibrium constant and q_m (mmol g^{-1}) is the amount of adsorbate required to form an adsorbed monolayer. K_F (mmol $L^{1/n}$ g^{-1} $mmol^{-1/n}$) and n are two Freundlich constants: n represents the relative advantage of adsorption process; K_F is the affinity constant which is related to the adsorption capacity of the adsorbent and can also be defined as adsorption or distribution coefficient, indicating the amount of PNP adsorbed on GO-ATT composites at equilibrium.

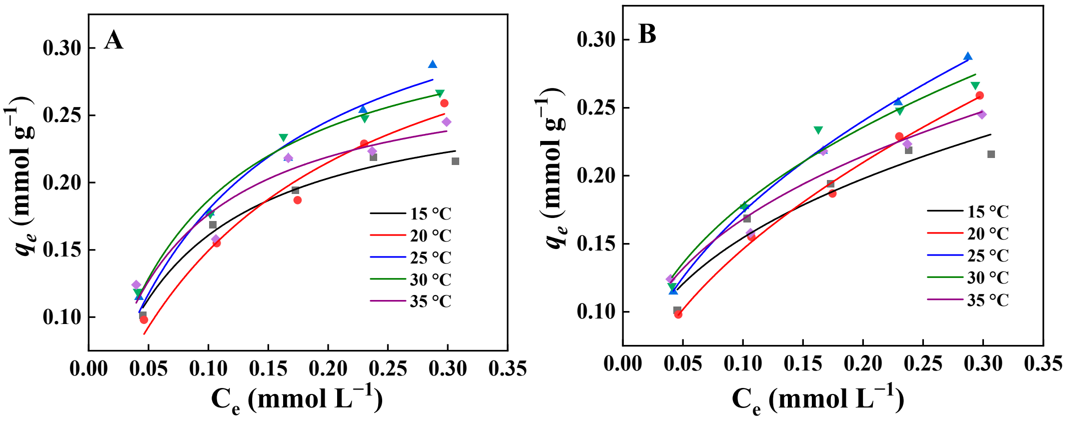

Fig. S2 (A) The fitting curves of Langmuir isotherm model; (B) The fitting curves of

Freundlich isotherm model.

Table S2 Adsorption isothermal parameters for the adsorption of PNP onto GO-ATT composites.

Model	Fitted parameter	15 °C	20 °C	25 °C	30 °C	35 °C
Langmuir	R^2	0.98191	0.97462	0.97866	0.98388	0.91851
	K_L (L $mmol^{-1}$)	13.96508	6.35866	8.6582	12.15686	15.55607
	q_m(mmol g^{-1})	0.27567	0.38379	0.38747	0.34017	0.28937
Freundlich	R^2	0.92505	0.99397	0.99856	0.97249	0.95042
	K_F (mmol $L^{1/n}$ g^{-1} $mmol^{-1/n}$)	0.35162	0.48668	0.51256	0.44715	0.37771
	n	2.79306	1.91033	2.12292	2.50973	2.83905

The thermodynamic parameters enthalpy change (ΔH^{θ}, KJ mol^{-1}), entropy change (ΔS^{θ}, J K^{-1} mol^{-1}) and Gibbs free energy change (ΔG^{θ}, KJ mol^{-1}) can be calculated by the following equations:

$$k_d = \frac{C_0 - C_e}{C_e} \times \frac{V}{m} \quad \textbf{(S8)}$$

$$\ln k_d = \frac{-\Delta G^o}{RT} = \frac{\Delta S^o}{R} - \frac{\Delta H^o}{RT} \quad \textbf{(S9)}$$

$$\Delta G^o = \Delta H^o - T\Delta S^o \quad \textbf{(S10)}$$

where k_d (L mg) is the thermodynamic equilibrium constant; V (L) and m (g) are the volume of adsorbate and mass of adsorbent, respectively; T (K) is the absolute temperature; and R (8.314 J mol^{-1} K^{-1}) is the ideal gas constant.

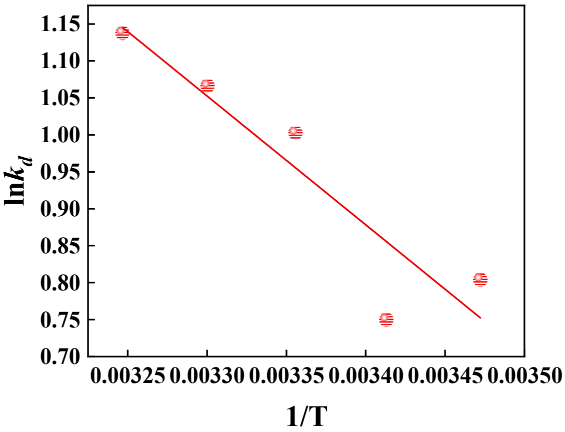

Fig. S3 Experimental data and fitted curves of lnk_d versus 1/T calculated from the van der Waals diagram of GO-ATT composites adsorbed PNP.

Table S3 Thermodynamic parameters of the adsorption of PNP onto GO-ATT composites.

T(K)	Δ*G*° (kJ mol^{-1})	Δ*H*° (kJ mol^{-1})	Δ*S*° (J K^{-1} mol^{-1})
288	-1.8017		
293	-2.0846		
298	-2.3675	14.492	56.5754
303	-2.6503		
308	-2.9332		
